# Editorial: Advances in natural product chemistry: Yunnan University 100th anniversary

**DOI:** 10.3389/fchem.2023.1234211

**Published:** 2023-06-13

**Authors:** Wen Chen, Jianwei Dong, Siva S. Panda

**Affiliations:** ^1^ Key Laboratory of Medicinal Chemistry for Natural Resource, Ministry of Education, Yunnan Provincial Center for Research and Development of Natural Products, Yunnan Characteristic Plant Extraction Laboratory, School of Pharmacy, Yunnan University, Kunming, China; ^2^ Colleage of Chemistry and Environmental Science, Qujing Normal University, Qujing, China; ^3^ Department of Chemistry and Physics, Augusta University, Augusta, GA, United States

**Keywords:** natural products, isolation and structural characterization, synthetic method, total synthesis, medicinal chemistry

Natural products have played important roles in drug discovery and development as more than 60% drugs are associated with natural products (Newman and Cragg, 2020). Yunnan University (YNU) located in Yunnan Province, China, the kingdom of plants, was founded in 1922 and officially opened in 1923. With this unique regional advantage, many interesting achievements in natural products have been made at YNU. To celebrate the 100th anniversary of YNU, this Research Topic aims to collect the latest developments in Natural Product Chemistry from school fellows of YNU, as well as the researchers who work or are involved in the development of YNU.

This Research Topic presents a Research Topic of reviews and original research articles on isolation and structural characterization of novel natural products (Chen et al., Lei et al., Yan et al., and Clements-Decker et al.), new synthetic approaches towards the key units of natural products (Rao et al. and Jiang et al.), total synthesis of natural products (Wei et al. and Wei et al.), and structure-activity-relationship (SAR) studies on bioactive natural-product-like molecules (Zhou et al., and Yin et al.). Taken overall, 10 contributions including 2 reviews and 8 original research articles comprise this Research Topic.

Chen et al. reported twelve new guaianolide sesquiterpene lactones, along with ten known analogs were isolated from an EtOH extract of the dried aerial parts of *Artemisia vulgaris* L. The isolated sesquiterpenoids dose-dependently exhibited an NO production inhibitory activity, which is better than that of the positive control (dexamethasone). A study on antioxidant and anti-inflammatory activity of constituents isolated from *Dendrobium nobile* (Lindl.) by Lei et al. shows that nineteen compounds, including two new vitamin E homologues, one new sesquiterpene, and two new dendrobines were isolated. New compound aldehyde-*α*-tocopherol demonstrated significant antioxidant activity compared with ascorbic acid (VC), as well as equal cytotoxic effect against Hela cell lines to cisplatin, indicating its potential application in the pharmaceutical and food industries. Yan et al. found fourteen C_19_-lycaconitine-type diterpenoid alkaloids, including six new alkaloids, grandiflolines A–F, isolated from *Delphinium grandiflorum* L. New alkaloids grandiflolines A-C and E possess a characteristic Δ^2,3^ functional group in the A ring, while grandiflolines E and F feature a rare OH-16 substituent. New alkaloids exhibit potential inhibition activities of NO in LPS-activated RAW 264.7 macrophages. A review conducted by Clements-Decker et al. delved into the antimicrobial properties of lipopeptides derived from various bacteria strains. The study sheds light on the structures and recently discovered frameworks of lipopeptide families produced by these bacteria, which possess promising antimicrobial properties. Furthermore, utilizing the genome mining approach, underexplored sources of novel antimicrobial lipopeptides have been uncovered. A detailed understanding of the mode of action and biosynthesis included in the review provides a clear path for the development of potential antimicrobial therapeutics in the future.

Rao et al. reported a new transitionmetal-free direct oxidative cyclocondensation reaction. The protocol highlighted the use of readily available *o*-aminobenzyl alcohols and *N*,*N*-dimethyl enaminones as starting materials, thus provided a flexible strategy for the preparation of 3-substituted or 3,4-disubstituted quinolones with broad substrate scope in moderate to excellent yields. The strategy enriched the quinoline synthesis method. Jiang et al. reported an effective palladium-catalyzed method for the synthesis of aryl acrylonitriles. This process uses simple and readily available arylacetonitriles and vinyl halides/triflates as raw materials, and the resultant aryl acrylonitriles can undergo a series of useful conversion reactions, such as reduction, hydrolysis and epoxidation.

Wei et al. reported a general strategy for the total synthesis of arylnaphthalene lactone lignans (NALLs) including justicidins B and E and taiwanin C. Key features of this synthesis include an aryl–alkyl Suzuki cross-coupling, a novel intramolecular cation-induced cyclization, and a base-mediated oxidative aromatization. Wei et al. explored the uses of dioxinones in synthesizing macrocyclic natural products and terpenoids. The review highlights the versatility and efficiency of dioxinones as reactive intermediates, making them valuable tools for the synthesis of diverse and complex natural products.

Zhou et al. designed and synthesized a series of anti-ZIKV active compounds, acetylarylamine-*S*-DACOs, and conducted in-depth research on the action mechanism of the representative compound through molecular docking analysis and a series of biological experiments. The results confirmed the anti-ZIKV activity at the molecular and protein levels, and discovered that this selected compound targeted ZIKV RNA-dependent RNA polymerase (RdRp). Yin et al. created a series of podophyllotoxin derivatives containing nitrogen-containing heterocycles, which may have potential as anticancer drugs. After synthesizing several derivatives, imidazolium salts and triazolium salts were found to be the most effective against different types of human tumor cells. Additionally, experiments on cell cycle and apoptosis revealed that these compounds could trigger G2/M cell cycle arrest and apoptosis in HCT-116 cells.

We hope that this Research Topic could provide an opportunity to school fellows of YNU to introduce their recent research findings in natural product chemistry to the chemical community, which could also provide an incentive for further scientific collaborations between school fellows of YNU and other researchers in this field.
